# Exploring Systemic Challenges in the Western North Carolina Dental Safety Net: A Qualitative Analysis

**DOI:** 10.13023/jah.0801.04

**Published:** 2026-04-01

**Authors:** Blaire Mirmow, Bill Milner, Nadia Laniado

**Affiliations:** Jacobi Medical Center; Access Dental Care; Jacobi Medical Center

**Keywords:** Appalachia, health care workforce, health disparities, oral health, public health, rural health

## Abstract

**Introduction:**

Despite statewide growth, rural communities in Western North Carolina (WNC) face persistent barriers to oral health care driven by chronic dental workforce shortages and systemic geographic disparities. Within this Appalachian region, the dental safety net serves as a vital yet strained support system, struggling with limited financial resources and a lack of provider participation that leaves many underserved populations without essential oral health services.

**Purpose:**

This study aims to explore the experiences of individuals working within the WNC dental safety net to identify barriers and strategies required to strengthen the delivery of oral health care in high-need rural settings.

**Methods:**

Semi-structured interviews were conducted with 17 oral health stakeholders involved in the WNC dental safety-net system between June – August 2024. Interviews explored challenges to meeting community oral health needs and potential solutions. Data were analyzed using thematic analysis.

**Results:**

Three major themes emerged: (1) workforce challenges involving dentists, hygienists, and dental assistants, (2) fragmentation between dental providers and the broader health care system, and (3) financial instability. Proposed solutions corresponding to each theme included streamlined credentialing and expanded allied health training programs to address workforce shortages, skills-based continuing education for public health dentists to reduce specialty referral gaps and fragmentation, and increased Medicaid reimbursement rates paired with coordinated policy advocacy to strengthen financial sustainability across the safety net.

**Implications:**

These findings suggest structural challenges facing rural safety-net programs and highlight opportunities to strengthen their capacity to meet growing community oral health needs.

## INTRODUCTION

Since the 2000 Surgeon General’s Report on Oral Health, the United States (U.S.) has made measurable progress in access to dental care.^1^,^2^ A substantial portion of this care is delivered through the dental safety net, a network of providers, facilities, and financing programs designed to support underserved and marginalized populations. This system functions as a critical lifeline for individuals who might otherwise be unable to obtain needed dental services. However, despite overall improvements, significant disparities in access to dental care persist.^1^ An estimated 72 million people in the U.S. lack dental coverage, nearly three times the number without medical insurance.^3^ Although dental safety-net programs serve as a primary access point for underserved populations, rising demand, driven by systemic inequities and Medicaid expansion, has outpaced available capacity in many community-based settings.^4^–^7^

North Carolina (NC) in particular has very concerning patterns of dental provider participation and dental service utilization, where only 31% of dentists participate in Medicaid and only 15% of Medicaid-insured adults are utilizing dental services.^8^ These challenges are particularly acute in rural areas, where longstanding workforce shortages and structural barriers to dental care have been further exacerbated by the COVID-19 pandemic.^9^ During the pandemic, thousands of dental hygienists exited the workforce, many of whom have not returned, placing additional strain on already limited workforce capacity.^9^ Approximately two-thirds of U.S. counties designated as dental health professional shortage areas (dHPSAs) are in rural communities.^10^ In Western North Carolina (WNC), a predominantly rural, Appalachian region, all 23 counties are designated as dHPSAs.^11^ With the state’s rapid population growth, the overall number of dentists in NC continues to grow, but the majority are concentrated in urban parts of the state, leaving rural communities with persistent shortages and growing disparities in oral health outcomes.^12^–^14^

Appalachian communities experience disproportionately higher rates of oral cancer and tooth loss compared to non-Appalachian communities.^15^, ^16^ The region’s oral health disparities reflect distinct challenges faced by Appalachian residents, including the long-term consequences of dental and food deserts.^17^ However, public discourse surrounding oral health in Appalachia frequently frames poor oral health as the result of individual behaviors rather than the structural barriers that shape access to care.^17^ This reinforces the importance of examining the systemic factors driving persistent oral health disparities in regions such as WNC. This study aims to explore the experiences of individuals working within the WNC dental safety net to identify barriers and strategies required to strengthen the delivery of oral healthcare in high-need rural settings.

## METHODS

### Study Design

This study used a qualitative descriptive design to explore the experiences of individuals involved with the oral health safety-net system in WNC, allowing for examination of the complex structural barriers shaping dental care delivery in the region. The study was approved by the Albert Einstein College of Medicine Institutional Review Board. We used the COnsolidating criteria for REporting Qualitative research (COREQ) checklist as a guide for reporting this study.^18^

Participants were eligible to participate if they were currently involved in efforts to improve access to oral healthcare services within the dental safety net across the 23-county region of WNC. Participants were recruited by the first (BM) and second (BEM) authors, who aimed to capture perspectives of different ages, genders, roles, counties, and levels of experience. A purposive sampling approach was utilized to identify oral health stakeholders with a wide range experiences including clinical leadership, administration, frontline care, and policy. Snowball sampling was also used as individuals were identified through professional networks and word-of-mouth referrals.

### Reflexivity

The first author (BM) is a dentist with experience working in public health settings across WNC. This study was completed as a University of North Carolina (UNC) Master of Public Health project in partnership with Access Dental Care (ADC), a North Carolina non-profit utilizing mobile dental services to treat special care populations across the state. The second author (BEM) is a dentist and president of ADC. Both BM and BEM had established relationships with some study participants prior to the study commencement.

To mitigate potential bias and ethical complexities, participants were informed in both written and verbal formats of the project’s connection to UNC and ADC, its goals, and the voluntary nature of their participation in the study. The third author (NL), a dentist in a safety-net hospital in New York City, brought an external perspective, critically examining the team’s interpretations for assumptions shaped by insider familiarity and regional positionality.

### Data Collection

Data collection was conducted after informed consent through one-on-one semi-structured interviews conducted both virtually and in-person between June – August of 2024. These interviews lasted approximately one hour each and were audio-recorded for later transcription and analysis. All interviews were conducted by one trained researcher (BM) to ensure consistency across data collection. The semi-structured interview guide explored participants’ professional challenges, organizational structures, and capacity to meet community oral health needs.

Thematic saturation was assessed through iterative review of transcripts as data collection progressed. Researchers independently evaluated whether new interviews were introducing novel codes or themes, discussing discrepancies until consensus was reached. Saturation was determined at approximately the 15^th^ interview when consecutive interviews produced no new codes and existing themes were consistently represented across participant accounts.

### Data Analysis

Data analysis followed the six-phase framework of reflexive thematic analysis.^19^ Two researchers independently reviewed the transcripts to achieve familiarity with the data and to develop an initial coding framework, which was refined through iterative discussion. Researchers negotiated toward consensus when coding disagreements arose. Excel was used to organize the data. Themes were generated through synthesis of coded data and examination of patterns across participant roles and settings. Ongoing analysis to refine themes and contextualize the findings occurred throughout the study. Peer debriefing was integrated throughout the analysis to strengthen rigor, reduce bias, and confirm that emerging themes were grounded in participant experience rather than researcher preconception.

## RESULTS

Seventeen oral health stakeholders (six men and 11 women) participated in the study, bringing between one and 20 years of professional experience in their respective roles. To capture a broad range of perspectives, participants were drawn from diverse institutions, including Federally Qualified Health Centers (FQHC), non-profits, health departments, private foundations, hospitals, state programs, and dental schools. Their efforts help to provide dental services across 18 of the 23 WNC counties.

Table 1 presents the three main themes related to challenges in the WNC dental safety net: (1) workforce recruitment and retention, (2) fragmentation across the dental and broader health system, and (3) financial instability, along with potential solutions.

### 1. Workforce

#### Dentist recruitment and retention

Participants described persistent difficulties recruiting dentists, dental hygienists, and dental assistants, citing compensation disparities with private practices as a primary barrier. As these wage differences widen, safety-net programs face increasing challenges in maintaining an adequate workforce. Participants noted that dental service organizations (DSOs), which offer high starting salaries and bonuses, are increasingly attractive to new dental graduates carrying substantial student loan debt. Although safety-net programs may appeal to job seekers through loan repayment options, participants stated that applicants were often deterred by limited opportunities to perform advanced dental procedures using new technologies emphasized in training, but less frequently reimbursed by public dental insurance programs.

Participants also reported that administrative burden and lengthy credentialing processes create delays in onboarding new dentists and maintaining participation across multiple insurance networks. Rural location was frequently cited as an additional barrier to recruitment. Concerns about housing availability, school quality, and limited recreational opportunities were described as deterrents for potential hires. While some dentists commute from nearby towns or cities, participants reported that lengthy commutes can become unsustainable over time.

Despite these challenges, participants reported that safety-net dentistry can offer meaningful professional benefits. Generous paid time off, flexible scheduling, and mission-driven work environments were described as motivating factors for clinicians committed to serving underserved populations. Participants emphasized that when organizations successfully recruit mission-aligned staff and provide supportive work environments, clinicians often build long-term careers in safety-net settings.

#### Hygienist and assistant recruitment and retention

Participants reported that dental hygienists are the most difficult dental workforce members to recruit, leading to gaps in essential preventive services. Participants explained how many hygienists did not return to work after the COVID-19 pandemic, and rising wages amid a limited labor pool have left safety-net programs struggling to find and afford needed staff. According to participants, safety-net organizations’ limited funding and fixed fees hinder competition with private offices, making workforce recruitment difficult. Recruitment of dental assistants was also reported as challenging due to a limited pipeline of trained personnel, particularly in rural areas. Some clinics responded to this deficit by offering on-the-job training, which they reported can be time consuming with slow productivity and result in high turnover, but can be beneficial with the right team member.

Participants explained that persistent staffing shortages since the pandemic have increased workloads, lengthened wait times, and reduced clinics’ ability to accept new patients. Participants reported that existing staff often absorb additional responsibilities, contributing to burnout and operational strain. Participants also emphasized the impact a quality front desk team can make on scheduling, collections, no show rates, and patient satisfaction. However, these roles often experience high turnover due to relatively low compensation.

### 2. Fragmentation

#### Between providers

Participants described a disconnect between private dental practices and safety-net clinics. Participants consistently reported that relatively few private practitioners in their communities accepted Medicaid, concentrating publicly insured and uninsured patients within safety-net clinics. Participants explained how safety-net programs, which are mission-driven organizations and provide care to all patients seeking treatment, struggle to keep up with the growing oral health needs in their communities.

Participants reported that some private practitioners are also leaving commercial insurance networks due to administrative burden and reimbursement concerns. Participants have noticed this shift has led more privately insured patients to seek care in safety-net clinics. While this modestly improves payor mix, participants reported it further strains already limited clinical capacity. Balancing care for new and existing patients was described as a persistent challenge. Participants reported that many clinics limit new patients to emergency services while maintaining long waitlists for comprehensive care.

Limited referral options for specialty care were also widely reported. Participants reported that patients often must travel 1–2 hours for specialty care, creating transportation and employment barriers, leading many to forgo treatment. Access to operating room dental services, particularly for the uninsured, is also increasingly scarce. Participants highlighted the critical role of dental school clinics within the safety-net. While these programs provide high-quality and accessible care, participants noted that longer student appointment times constrain their capacity to meet community needs.

#### Between systems

Participants also described a disconnect between dentistry and the broader health system. Many reported that organizational leadership within safety-net institutions is often composed primarily of medical administrators with limited familiarity with dental care delivery, coding, and reimbursement structures. Participants explained that when funding is not specifically earmarked for oral health, resources are often directed toward medical departments. Participants also reported challenges connecting patients with extensive social needs to community resources, which can complicate dental care delivery. Despite these challenges, participants noted that integrated models, such as co-located services and shared electronic health records, can improve communication and care coordination.

### 3. Financial instability

Participants commonly expressed concern about the financial sustainability of safety-net dental programs under existing structures, which rely primarily on patient revenue and grants from public and private sources. Participants explained that while grants are helpful, they can be unreliable and typically only fund startup costs for specific projects, without long-term sustainability. Most participants reported their programs were operating at a financial loss. Nearly all participants identified low Medicaid reimbursement rates as a critical barrier, limiting progress toward the widely shared goal of operational self-sufficiency. Participants emphasized that stagnant reimbursement structures make it increasingly difficult to maintain services while meeting workforce and operational needs.

Participants also reported that current reimbursement models often fail to account for the complexity of patients served in safety-net settings. Many patients require longer appointments, care coordination, or additional support services that are not reflected in billing codes. Participants working in FQHCs described challenges associated with the Prospective Payment System (PPS), which provides a bundled per-visit reimbursement regardless of the number of procedures performed. Participants explained that this structure may unintentionally encourage multiple visits and does not always align with efficient delivery of dental care, particularly for patients who face complex social needs.

Participants highlighted the inherent tension between maintaining a sustainable payor mix and upholding the mission to provide care regardless of a patient’s ability to pay. At FQHCs operating under sliding fee scales, participants reported that most patients qualify for the lowest payment tier and collection rates are often low. Although Medicaid expansion has increased coverage, participants emphasized that North Carolina Medicaid dental reimbursement rates have remained frozen since 2008, limiting the financial benefits of expanded coverage.

Participants also reported trying to keep their programs afloat by increasing their pediatric patient pool, often through school-based programs, due to higher reimbursement for pediatric dental services. Challenges include sustainably treating children who are uninsured. Participants highlighted the positive impact of the 2020 Public Hygiene rule revision, which now allows public health hygienists to practice in certain settings in dental shortage areas without a dentist on site.^20^

Potential solutions to these challenges are presented in Table 2 and correspond to the three major themes of the study.

### 1. Workforce solutions

Participants emphasized the need to streamline NC’s dental credentialing process to reduce delays in hiring new providers. Expanding dental hygiene and assisting training programs in rural areas was also frequently suggested. Participants described partnerships with community colleges and allied health programs as effective strategies for building workforce pipelines.

### 2. Fragmentation solutions

Participants suggested additional training opportunities, such as mini-fellowships or other continuing education programs, could expand the scope of services offered by safety-net dentists and reduce reliance on specialty referrals. Participants also described using patient demand indicators, like waitlists, to communicate workforce and capacity needs to organizational leadership.

### 3. Financial instability solutions

Participants recommended increasing Medicaid reimbursement rates to match an annual national benchmark to keep up with inflation. Participants also emphasized the importance of strengthening advocacy and representation of safety-net dentists in policy discussions affecting reimbursement and regulatory frameworks on the local, state, and national levels.

## IMPLICATIONS

This study documents the lived realities of safety-net dentistry in WNC, reflecting challenges that, while regionally focused, are emblematic of broader statewide and national trends.^8^ Participants consistently described a system under strain, suggesting that current funding and delivery structures are insufficient to sustain equitable access to dental care for underserved populations in rural Appalachian communities.

Fragmentation emerged as a central theme across participant accounts and operates at multiple levels. Structurally, fragmentation reflects differences in payment systems, insurance participation, and incentives across private practices, safety-net clinics, and the broader health care system. Culturally, it reflects longstanding separation between medical and dental professions, and limited familiarity with dental care delivery among health system leadership.^21^ These dynamics can hinder collaboration and limit integration of oral health within broader health initiatives.

Participants also emphasized the central role of Medicaid reimbursement in shaping the sustainability of safety-net dental programs. Across both fee-for-service and the PPS systems, participants consistently reported that reimbursement levels do not adequately cover the cost of providing care for medically and socially complex populations. Research has documented similar trends nationally, where Medicaid dental reimbursement rates fall significantly below private insurance rates and provider participation remains limited.^8^ Meaningful participation by private practitioners in Medicaid networks will require reimbursement reforms that make serving this population financially viable. Following the lead of states like Utah and Oregon by expanding Medicaid reimbursement to include codes that address appointment barriers (D9991), care coordination (D9992), and case management for patients with special health care needs (D9997) could encourage greater provider participation for high-needs populations.^22^

Participant experiences highlight how workforce shortages across multiple dental roles constrain clinic capacity, limiting the ability of safety-net programs to expand preventive and comprehensive services in rural areas. In Appalachia, where the terrain is rugged and mountainous, weather-related road closures, increased isolation, and limited accessibility to a small pool of providers make accessing needed services particularly challenging.^23^ Expanding the workforce by integrating preventive oral health services into primary care settings may help to expand access.^24^

Two months after the conclusion of these interviews, Hurricane Helene devastated WNC, resulting in nearly $60 billion in damages statewide.^25^ This historic storm disrupted health services, damaged healthcare infrastructure, and exacerbated housing and economic instability across the region.^25^ Future research should examine how this event affected the resilience of dental safety-net programs serving rural Appalachian communities.

A strength of this study is the diversity of participants representing multiple institutions, professional roles, and geographic areas within the region. However, several limitations should be noted. Although many of the experiences described may resonate with stakeholders in other rural settings, caution is warranted when transferring these findings beyond NC due to variation in Medicaid policies and safety-net structures across states. Additionally, the purposive and snowball sampling approach may have favored individuals more engaged in regional safety-net networks. Lastly, the researchers’ pre-existing professional relationships in the region may have influenced participant disclosure and thematic interpretation.

Ultimately, the findings suggest that the current dental safety net in WNC is operating under increasingly unsustainable conditions. While participants described strong mission-driven commitment and local innovation, structural constraints related to workforce supply, payment systems, and care coordination continue to limit system capacity. Without coordinated policy reforms and sustained investment, safety-net programs will remain constrained in their ability to meet community needs and reduce oral health inequities in rural Appalachia.

**SUMMARY BOX**

**What is already known about this topic?**

Rural populations experience increased barriers to dental care, evidenced by worse oral health outcomes. Many in these communities rely on the dental safety-net for their care which is often under resourced and faces systemic barriers to meeting community oral health needs.

**What is added by this report?**

This study adds qualitative, place-based evidence to the oral health literature by documenting how workforce shortages, system fragmentation, and chronic under resourcing shape the day-to-day functioning of dental safety-net programs in rural Western North Carolina.

**What are the implications for future research?**

Future research should utilize the insights from oral health stakeholders in this study to evaluate how targeted workforce, training, and Medicaid policy interventions influence the capacity, integration, and sustainability of rural dental safety-net programs across diverse state and regional contexts.

## Figures and Tables

**Table 1:** Systemic challenges in the Western North Carolina dental safety net – Summary quotes

Theme #1: **Workforce**	
*Dentist recruitment and retention*	If you’re hiring a new dentist, they can’t start until they’re credentialed, so we’re losing some of our new dentists to other states because they can get hired more quickly in South Carolina or Tennessee or Georgia than they can here, like six months earlier. *-Grant officer*
	It is just hard to recruit people to come work out here. I commute, and it’s not sustainable long term. *-Hospital dental director*
*Hygienist and dental assistant recruitment and retention*	I have no hygienist. My hygienist left to take another job that paid more money. I have positions for 2 full-time hygienists. I’m trying to get one part-time hygienist, and it has to do with state funds. How much, we’re a state organization and what the state can pay. Are we competitive? Especially for hygienists? And the answer is, no. *-Dental school faculty*
	If you tour X’s site, they have like 2 dental assistants for 16 student dentists. So, if you think of a regular dental office where they might have 4-6 dental assistants per dentist, the ratio is way flipped on its head. *-Grant officer*
	I think that’s part of the reason that we have such a hard time with providers and assistants and front desk is because if you’re going to work every day and you’re in a war against decay and against oral health diseases, it’s exhaustive right now. You feel like there’s no help and there are never enough hands. *-FQHC dental director*
	At our last dental director meeting, we were all just kind of shaking our head like, what are we going to do if we can’t pay our folks more? *-FQHC* d*ental Director*
Theme #2: **Fragmentation**	
*Between providers*	Both of those (private) practices have been sold and now it’s run by a corporation. When that organization came in, they basically just stopped taking Medicaid and then we had a huge influx of patients trying to get care. *-Health department dental director*
	I do feel like (managing treatment plans) has gotten a lot better since we have closed the wait list, reduced the number of new patients on the schedule, but it’s such a tight rope to walk right? Because at the same time we’re getting so many calls. People have Medicaid now. They can’t find anybody to take them and so the demand is always there. It became an occupation like it became a job description managing the wait list. *-FQHC dental director*
	I have to beg or borrow a lot of times to get somebody to see Medicaid patients, even though they may say we see them. And then, when you call, it’s a fight. So, it becomes very difficult to see some of these patients or to refer some of these patients. And a lot of these private practitioners are falling into different groups, specialty groups, so it becomes very hard to reach out and touch base to say I need your help. And you know, if I call, I really need to have help. *-Dental school faculty*
*Between systems*	Dentistry kind of sits on this island whether they’re in a health department or an FQHC and if administration doesn’t understand the differences, they can’t help support them. *-FQHC dental director*
	I hear all the time that we have so many resources, but the coordination of it... it is a major problem. We have all these resources, but the clients can’t navigate that system. *-Non-profit leadership*
Theme #3: **Financial instability**	
I feel that the greatest concern that I’ve seen from the various FQHCs that I’ve worked with is always the financial and the sustainability aspect. *-FQHC dental director*	
If (Medicaid reimbursement) doesn’t even pay lab costs for a partial denture then you’re operating in red all the time. *-State administrator*	
They can’t afford to serve folks because everyone is taking a loss on oral health right now. And so really, I shouldn’t advocate too much more for these vital safety-net organizations to increase dental because I don’t want them to go under. -*Grant officer*	
Some of those basic needs, we can’t do volume, just from a billing standpoint if we’re having to do all this extra, I call it the social work of dentistry, coordination, and we get paid for none of that. *-Non-profit leadership*	
How can you survive on rates that were prevailing 15 years ago? The answer to that question is, you can’t. You can’t without cost shifting to other payer sources. *-FQHC dental director*	

**Table 2:** Potential solutions to systemic challenges – Summary quotes

Theme #1: **Workforce**	
*Streamline credentialing*	Every other state has it worked. It’s written into law that the managed care organizations have to credential providers within 90 days of a full and complete application. We don’t have such standards for our managed care organizations and so, some of them want to credential earlier, but they’re actually not even held accountable. Other states say you have to do NCQA to the National Committee and Quality Assurances credentialing standards like so. It’s all laid out. So, it makes it easier for managed care to do credentialing too. *-Grant officer*
*Align training programs and partnerships with workforce needs*	If you look at where the dental assistant schools are, there are some dental assistants, right? Is that where they are needed? *- FQHC dental director*
	Hygiene has been the most difficult out here, and we were fortunate that we snagged not one, but two AB tech grads, and that came out of one, a partnership that we developed with AB tech where we go up there and would we speak to their students, talk to them about opportunities and FQHCs and public health, particularly one that’s just down the road from where they’re at. And two, we also became a site, a rotation site for dental assisting students. *-FQHC dental director*
Theme #2: **Fragmentation**	
*Innovative continuing education programs*	...increasing the skills of your general dentist to tackle some of those trickier periodontal issues. There’s a large number of those things referred out that if general dentists can, with these mini-fellowships, gain expertise and skills, some of these more specialty procedures.. we can prevent having to have them done in the ER, which is near impossible sometimes because the hospitals don’t want to lose their high revenue operating rooms. And then how can you get that person surgery? So, like when you look in the West and you look at our Medicaid spending, we are the top in our in the state for unnecessary ER utilization for oral healthcare. I think we’re quadruple. *-Grant officer*
*Leverage patient demand metrics*	The value that I saw in keeping our wait list was, I was able to take that to our board of directors and say, I need more space. I need more resources, and that’s what yielded us a clinic that’s soon to open that’s triple the size of what I’ve currently got. *-FQHC dental director*
Theme #3: **Financial instability**	
*Increase North Carolina Medicaid reimbursement rates* *Increase advocacy efforts of dental safety-net stakeholders*	Being self-sustainable as a department could be made a whole lot easier if payers paid more. The other component that makes it difficult is just sheer volume and demand for services. It’s great to have patients beating down the door, but it can also be difficult to serve the patients that we’re serving just given various barriers that our patients may have in their lives which could contribute to either no shows or bad debt, or anything along those lines. And so that’s kind of one thing that we’re constantly juggling is meeting the need of the patients, meeting the demand for services, but also doing so in a way that’s going to ensure that we live to fight tomorrow. *-FQHC dental director*
	We have the system that we have designed and if we want a different system, we have to design and fund it. I would say the biggest part of system design is the funding mechanism and that’s extremely important. *-Grant officer*
	I think an increased voice or an increased presence of safety-net dentists on the state and national level is something that really needs to happen and advocate specifically for the way that we do dentistry and the way that dentistry is delivered in kind of this this setting. *-FQHC dental director*
